# Egg perivitelline fluid of the invasive snail *Pomacea canaliculata* affects mice gastrointestinal function and morphology

**DOI:** 10.7717/peerj.5314

**Published:** 2018-10-29

**Authors:** Matías Giglio, Cintia Garro, Enrique Caviedes-Vidal, Horacio Heras

**Affiliations:** 1 Facultad de Ciencias Naturales y Museo, Universidad Nacional de La Plata, La Plata, Argentina; 2 Instituto de Investigaciones Bioquímicas de La Plata (INIBIOLP), Consejo Nacional de Investigaciones Científicas y Técnicas y Universidad Nacional de La Plata, La Plata, Argentina; 3 Instituto Multidisciplinario de Investigaciones Biológicas de San Luis (IMIBIO-SL), Consejo Nacional de Investigaciones Científicas y Técnicas y Universidad Nacional de San Luis, San Luis, San Luis, Argentina; 4 Departamento de Bioquímica y Ciencias Biológicas, Universidad Nacional de San Luis, San Luis, San Luis, Argentina

**Keywords:** Apple snails, Intestinal enzymes, Intestinal absorption, Effect of perivitellin fluid, Intestinal morphology

## Abstract

**Background:**

Species beloging to the genus *Pomacea* (Ampullariidae), often referred as apple snails, are freshwater, amphibious snails native to South, Central and North America. Some species such as *P. canaliculata* have become a driver of ecosystem changes in wetlands and an important rice and taro pest after its introduction to Asia and other parts of the world. Females deposit colored egg clutches above the waterline, a reproductive strategy that exposes the eggs to harsh conditions and terrestrial predation. However, eggs have no reported predators in their native range, probably because of the acquisition of unparalleled biochemical defenses provided by a set of proteins (perivitellins) that nourish embryos and protect them from predators and abiotic factors. Notably, ingestion of egg perivitelline fluid (PVF) decreases rat growth rate and alters their gastrointestinal morphology. The aim of the study is to determine the effect of apple snail egg PVF on mice gut digestive activity, morphology and nutrient absorption.

**Methods:**

Carbohydrate digestion by intestinal disaccharidases (sucrase-isomaltase and maltase-glucoamylase) was evaluated *ex vivo* in mice gavaged with 1 or 4 doses of PVF. Changes in gut morphological and absorptive surface were measured. In addition, alteration on nutrient absorption rates, transport pathways and intestinal permeability was evaluated by luminal perfusions of small intestine with radiolabeled L-proline (absorbed by paracellular and transcellular pathways) and L-arabinose (absorbed exclusively by paracellular pathway).

**Results:**

Perivitelline fluid affected mice displayed significant morphological changes in the small intestine epithelium inducing the appearance of shorter and wider villi as well as fused villi. This resulted in a diminished absorptive surface, notably in the proximal portion**.** Likewise, the activity of disaccharidases diminished in the proximal portion of the intestine. Total absorption of L-proline increased in treated mice in a dose-dependent manner. There were no differences neither in the ratio of paracellular-to-transcellular absorption of L-proline nor in gut permeability as revealed by the clearance of L-arabinose.

**Discussion:**

Oral administration of apple snail PVF to mice adversely alters gut morphophysiology by reducing the intestinal absorptive surface, affecting enzymes of sugar metabolism and increasing the absorption rate of nutrients without affecting the relative contribution of the absorption pathways or gut permeability. These results**** further support the role of PVF in passive anti-predator defenses in *Pomacea* snail eggs that target the digestive system.

## Introduction

*Pomacea* (Caenogastropoda: Ampullariidae), commonly known as apple snails, are a genus of freshwater snails native to South, Central and North America (Hayes, Cowie & Thiengo, 2009; Hayes et al., 2012). In contrast to most aquatic gastropods, *Pomacea* species are amphibious, laying calcareous and conspicuously colored egg masses above the waterline (Heras et al., 2007; Heras et al., 2008), a reproductive strategy considered as a key acquisition associated with their successful diversification and dispersion (Hayes et al., 2009). Conversely, this reproductive strategy exposes the eggs to harsh conditions like sunlight, desiccation and terrestrial predators (Heras et al., 2007). To cope with that embryos are surrounded by a perivitelline fluid (PVF) that nourishes and protects them, mainly composed of polysaccharides and proteins (perivitellins) (Garín, Heras & Pollero, 1996; Giglio et al., 2016; Hayes et al., 2015; Heras, Garín & Pollero, 1998; Heras et al., 2007). The protective functions described for perivitellins include antioxidant, photoprotective, antiprotease, antinutritive and toxic (Dreon, Heras & Pollero, 2004; Dreon, Ituarte & Heras, 2010; Heras et al., 2008). The presence of noxious proteins is advertised by the egg conspicuous coloration (aposematic coloration) considered a warning signal to potential predators. This complex defensive system is very effective and in fact apple snail eggs are not known to have predators in their native range and only one, the fire ant *Solenopsis geminata,* in southeastern Asia where *P. canaliculata* snails has been introduced and become an invasive species (Yusa, Sugiura & Ichinose, 2000). Furthermore, common predators of adult snails, such as rats and snail kites, discard the albumen-capsule gland complex, the organ that synthesizes and stores the egg PVF (Cadierno, Dreon & Heras, 2017; Yusa, Sugiura & Ichinose, 2000).

As the epithelial cells along the digestive tract are fully exposed to food contents, they are a possible target site for defensive proteins. In this regard, plants have evolved a wide array of toxic dietary lectins that interact with the membrane glycoproteins of the luminal side of the gut of predators having an important role in plant seed defenses against predation (herbivory) (Peumans & Van Damme, 1995). In animals, however, similar embryo defenses have only been described in *Pomacea* spp. (Dreon et al., 2014; Pasquevich et al., 2017). In fact, oral administration of *P. canaliculata* PVF to rats causes a decrease in its growth rate and induces large morphological changes in the small intestine mucosa which reduce its absorptive surface Furthermore, *P. canaliculata* PVF showed strong cytotoxic activity on Caco-2 cells in culture, indicating the presence of toxins somehow damaging enterocytes (Dreon et al., 2014). The antiprotease activity of the PVF from several *Pomacea* species have also been suggested as an antidigestive defense system (Giglio et al., 2016). Other digestive components that could be targeted by egg defenses are the membrane-bound digestive enzymes of enterocytes such as disaccharidases. However, there is no information on the effect of snail PVF on membrane-bound sugar digestion enzymes.

The intestinal absorption of water-soluble nutrients (e.g., monosaccharides and amino acids) is performed by one of two pathways: transcellular, the transporter-mediated absorption of nutrients through enterocytes, and paracellular where nutrients move passively through a small space between enterocytes (Pappenheimer & Reiss, 1987). These pathways do not contribute equally to the water-soluble nutrients absorption among vertebrates (Caviedes-Vidal et al., 2008; Fasulo et al., 2013; Lavin, McWhorter & Karasov, 2007). For instance, paracellular pathway accounts only for 20–30% of glucose and amino acids absorption in rodents (Brun et al., 2014; Lavin, McWhorter & Karasov, 2007; Price et al., 2014). There is no information on the effect of PVF on rodent nutrient absorption. We hypothesized that PVF induced alterations of small intestine morphology (Dreon et al., 2014) would be accompanied by changes of digestive physiology.

The aim of this study was to develop a better understanding of the defensive mechanisms of apple snail eggs through an examination of the effects of *P. canaliculata* egg extracts (PVF) on gut morphophysiology of potential predators using mice as a murine model. This model has been chosen considering rodents are among the few apple snails predators that avoid eating eggs, and discard the PVF-containing albumen-capsule gland complex (Yusa, Sugiura & Ichinose, 2000). In particular, we determined the effect of PVF on mice’s ability to digest and assimilate carbohydrates, intestinal permeability of water-soluble nutrients, and on the intestinal morphology and absorptive surface.

## Materials and Methods

### Ethics statement

All experiments with mice have been made according to institutional animal use regulations and approved animal use protocols by the Animal Care and Use Committee of the Universidad Nacional de San Luis (UNSL), protocol number B205/15, and were carried out in accordance with the Guide for the Care and Use of Laboratory Animals (National Research Council, 2011).

### Egg collection and perivitelline fluid preparation

Fresh egg masses of *P. canaliculata* were collected from females raised in our laboratory at Universidad Nacional de La Plata (UNLP) from a colony established with eggs from an artificial pond in La Plata, Argentina (34°54′38″S; 57°56′17″W). Clutches were rinsed with ice cold 20 mM Tris-HCl, pH 7.8, and homogenized in a Potter type homogenizer with a buffer: sample ratio of 3:1 v/w. The crude homogenate was then sequentially centrifuged at 10,000× g for 30 min in an Avanti JE centrifuge (J25-50 rotor) and at 100,000× g for 50 min in a Beckman L8- centrifuge (Ti 70.1 rotor). The pellet was discarded and the supernatant was considered the perivelline fluid (PVF). The total protein concentration of the PVF was determined by the method of Lowry et al. (1951).

### Mice

All experiments were performed using adult male and female BALB/C mice (*Mus musculus*) (28.3 ± 0.5 g; mean ± SEM). They were held in cages at relatively constant temperature (22 ± 2 °C), relative humidity of 35 ± 3%, and with a lighting schedule of 13:11 h light: dark. Animals had access to water and food *ad libitum*.

### Gavage

Absorption experiments and enzyme assays were performed with three groups of treated mice, 10 animals each (five males and five females). One group was gavaged with 300 µL of PVF fraction (560 µg total protein) 12 h before perfusion (one-dose group). Another group of mice were gavaged with doses of 300 µL of PVF fraction (560 µg total proteins) every 24 h during 4 days before perfusion (four-dose group). The control group (10 mice) received the equivalent volume of water. Oral gavage was performed using a winged needle infusion set and were completed within 30 s.

### Luminal perfusions

To examine tissue-level absorption, we used *in situ* intestinal luminal perfusions. The animals were anesthetized with isoflurane (1–5%) and oxygen delivered by a vaporizer (Surgivet/Anesco Isotec 4; Surgivet, Dublin, OH, USA). Mice body temperature during the procedure was maintained using a heating pad at 37 °C (Braintree Scientific Inc., Braintree, Massachusets). The abdominal cavity was opened with a peritoneal incision and proximal and distal ends of the intestine were identified. The intestine was cannulated at ∼1 cm from the stomach using a rat gavage needle as the enter cannula and an exit cannula was placed ∼10 cm away from the first one, both extremes were secured with sutures. The intestine was flushed with a pre-warmed saline solution (9%) to remove its contents. The saline solution was then evacuated with air. Finally the animal was perfused with a perfusion buffer containing 85 mM NaCl, 5 mM NaHCO_3_, 2.5 mM KCl, 1 mM MgSO_4_, 1 mM CaCl_2_, 10 mM D-glucose, 10 mM L-proline, 10 mM L-arabinose, using a perfusion pump (Watson-Marlow Alitea 400) during 2 h at a rate of 1 mL min^−1^. During the procedure the perfusate returned to a reservoir and was continuously recirculated. The solutions were labeled with a tracer amount of [1-^14^C]-L-arabinose and [2,3-^3^H]-L-proline (Perkin Elmer). The perfusate was weighted carefully before and after the perfusion. Subsamples (50 µL) of the perfusate collected before and after the perfusion were counted in 4 mL Ultima Gold TM scintillation cocktail (Perkin Elmer, Waltham, MA, USA) in 8 mL glass scintillation vials with a scintillation counter (Wallac 1409 DSA; Perkin Elmer, Waltham, MA, USA). At the end of the surgery the animal was euthanized with isoflurane at 5% monitoring pedal withdrawal and palpebral reflexes until animals were fully anesthesized. The intestine was dissected out and the length and circumference of the perfused segment was measured. After perfusion assay, the whole small intestine (i.e., perfused and non-perfused parts) was removed and divided in three portions of the same length named proximal, medial and distal. Samples of each portion were collected for the following assays.

Absorption of each probe was calculated from the decrease in total radioactivity during the experiment, normalized by the duration (min) of the perfusion and by nominal surface area (cm^2^) of the perfused section of intestine. For L-arabinose, the clearance was also calculated to account for the slight changes in probe concentration over the course of the experiment. To calculate clearance (µl min ^−1^ cm^−1^), we divided absorption rate by [(C_initial_ − (*C*_final_)∕ln(*C*_initial_∕*C*_final_))], where *C* stands for probe concentration (Sadowski & Meddings, 1993). Clearance values for L-proline were not calculated because it is absorbed by both carrier-mediated and non-mediated mechanisms.

Since L-proline is absorbed through both paracellular and transcellular pathways, its absorption represents the total absorption, while L-arabinose absorption is only passive and represents the paracellular absorption (Chediack et al., 2001). Using this information we estimate the proportion of paracellular and trancelullar nutrient absorption.

### Enzyme assays

The activity of two intestinal disaccharidases were measured: sucrase-isomaltase (E.C. 3.2.1.10) and maltase-glucoamylase (E.C. 3.2.1.3), which catalyze the hydrolysis of sucrose and maltose, respectively. After perfussion, tissue samples were stored at −70 °C until used. Enzyme activity was determined in control and treated animals in the three sections of the small intestine. We used the colorimetric method developed by Dahlqvist (1968) and modified by Martínez del Río (1990). Briefly, tissues were thawed at 4 °C and homogenized for 30 s in 300 mM mannitol in 1 mM N-2-hydroxyethylpiperazine-N′-2-ethanosulfonic acid (HEPES)-KOH, pH 7.0, keeping a 1:100 (v:w) buffer:sample ratio, using a manual homogenizer (Fisher Scientific™ Laboratory Homogenizer, Model 125; Waltham, MA, USA). The resulting homogenate was diluted 10 and 100 times for sucrose-isomaltase and maltase-glucoamylase assays, respectively. Aliquots of 40 µL of diluted intestinal homogenates were incubated with 40 µL of 56 mM sucrose or 56 mM maltose in 0.1 M maleate/NaOH buffer, pH 6.5, at 37 °C for 20 min. The activity of the enzymes was measured following the amount of hydrolyzed glucose using the “Glucosa Liquid plus” kit (GT Laboratorios SRL, Santa Fe, Argentina) following manufacturer instructions. Reactions were allowed to stand for 20 min at room temperature and the absorbance measured at 505 nm. Enzyme activity (µmoles min^−1^) was determined using a glucose standard curve.

### Histological and morphological measurements

Histology was performed following that described in Dreon et al. (2014). In short, after perfusion assay intestinal tissue samples from proximal, medial and distal portions were inmediatedly fixed in 10% neutral formaldehyde for 24 h, then dehydrated with ethanol and stored until processed. Samples were then completely dehydrated in ethanol 100% and embedded in paraffin wax. Sections (5–7 µm) were stained for general morphology analysis with hematoxylin and eosin.

Ten to fifteen villi and crypts from small intestine mucosae were selected at random and their length and width measured. This data was employed to calculate mucosal absorptive surface area following the method of Kisielinski et al. (2002). The surface area was calculated using mean values of the structures that define the mucosal unit, namely villus length and width, and crypt width. The mucosal-to-serosal amplification ratio *M* was calculated as follows:

\begin{eqnarray*}M= \frac{ \left( villous~width\ast villuos~length \right) +{ \left( \frac{villous~width}{2} + \frac{crypt~width}{2} \right) }^{2}-{ \left( \frac{villous~width}{2} \right) }^{2}}{{ \left( \frac{villous~width}{2} + \frac{crypt~width}{2} \right) }^{2}} \end{eqnarray*}

### Statistics

Statistical analyses were conducted with R statistical software and results are expressed as mean ± 1 SEM. The effect of PVF on parameters (i.e., enzyme activity, absorption, clearance and paracellular/transcellular ratio) was determined by one way analysis of variance (one way ANOVA) with post-hoc Tukey’s test. The *F*-values of these and other analyses of variance are presented in the text with the relevant degrees of freedom as subscripts. The significance level selected to accept difference for all statistical analysis performed was *α* <0.05.

## Results

### Effect of PVF on mice gastrointestinal morphology

**Figure 1:**
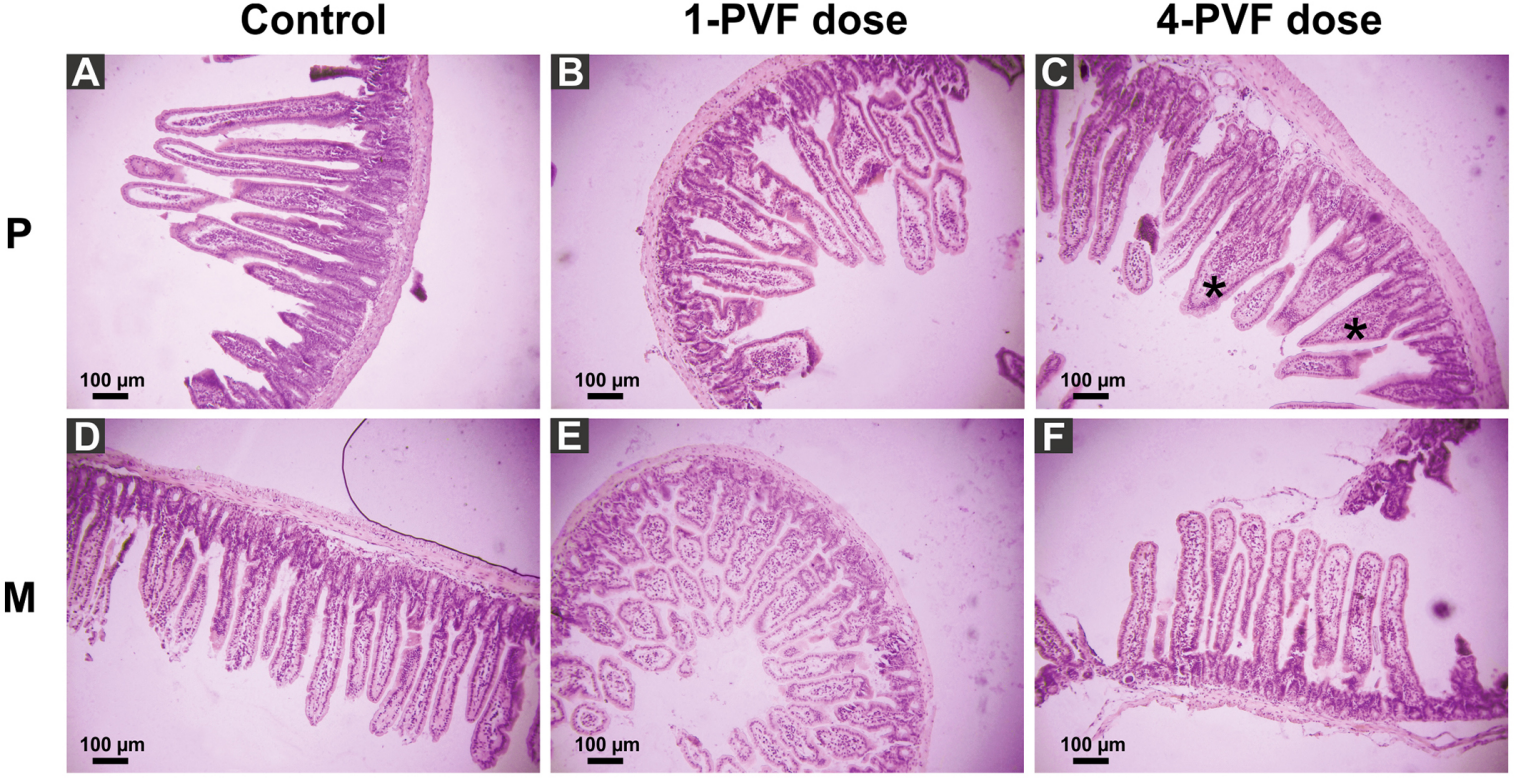
Effect of *P. canaliculata* PVF on mice small intestine morphology. Mice were fed on a diet without (A, D) or with (B, C, E, F) PVF containing 0.56 mg protein each dose. P: proximal section. M: medial section. * fused, “tonge-like” villi. Bar 100 µm.

**Figure 2:**
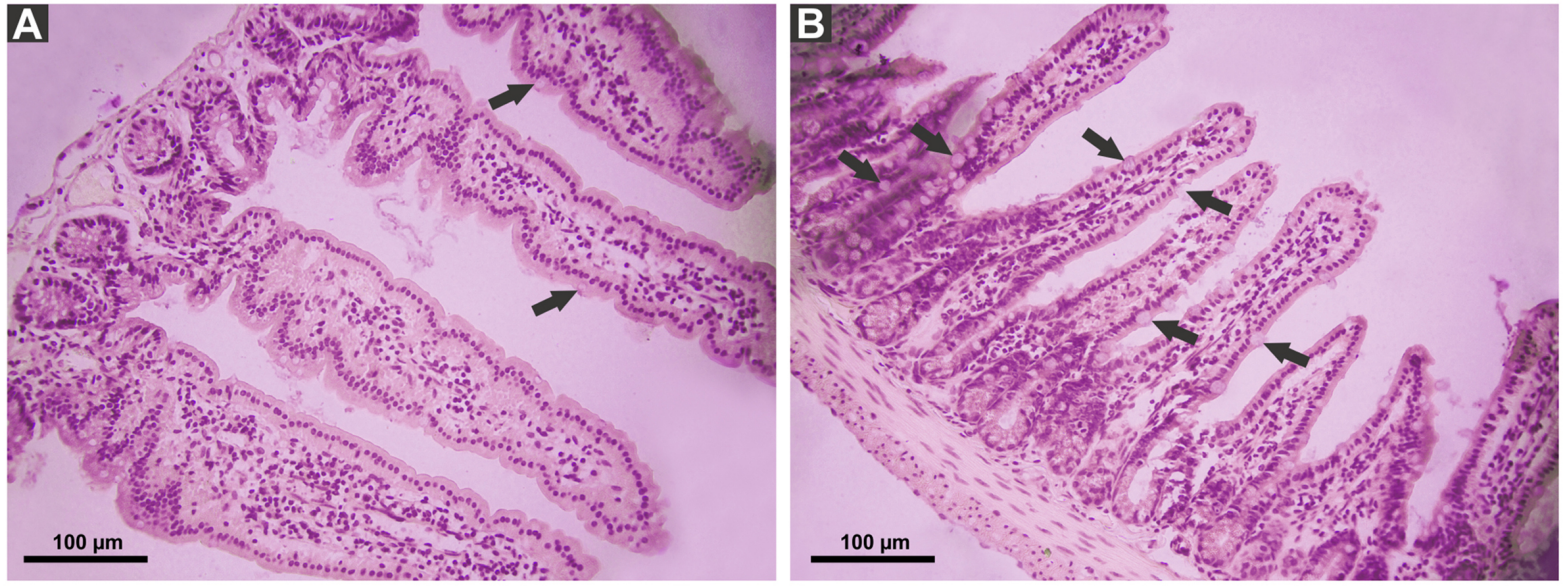
Effect of *P. canaliculata* PVF on goble cells. Mice were fed on a diet without (A) or with PVF (B) (four doses of 0.56 mg protein each) (B). Arrows indicate goblet cell. Bar 100 µm.

*P. canaliculata* PVF induced substantial morphological changes in mice small intestine. Most notably, villi from treated animals were shorter and wider than those of control mice, some with a “tongue-like” shape (Fig. 1, asterisks). In addition, an augmented number of goblet cells was observed in the mucosae of treated animals (Fig. 2).

**Figure 3:**
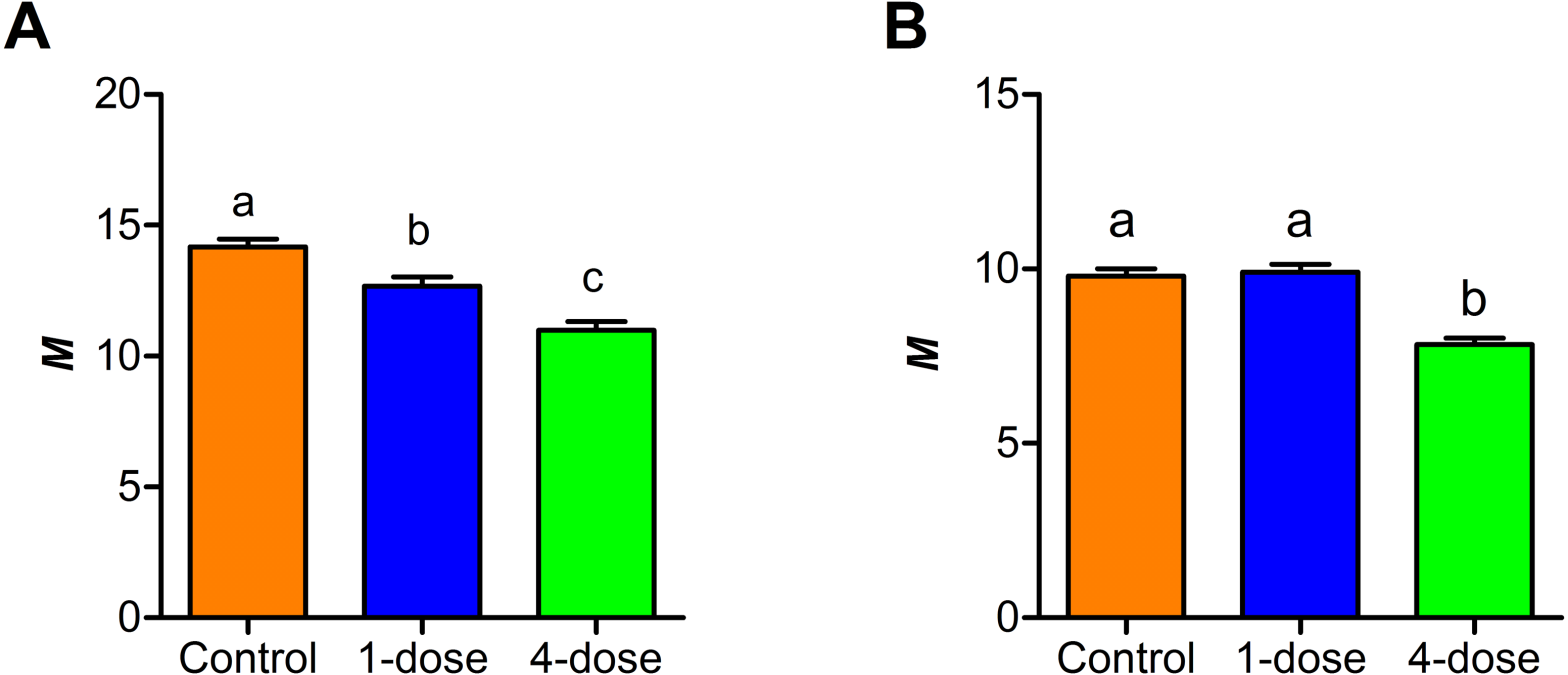
Changes in small intestine mucosal absorptive surface of mice gavaged with *P. canaliculata* PVF. Proximal (A) and medial (B) portions of the intestine. (*M*) Mucosal-to-serosal amplification ratio. Each PVF dose contans 0.56 mg protein.

A comparison between the Kisielinski parameter (*M*) of the proximal portion of intestines of the treated animals showed a decrease in the absorptive surface (*F*_2,227_ = 22.14; *P* < 0.001). The decrease was evident in animals exposed to a single dose of PVF (*P* < 0.01) and more markedly after four PVF doses (*P* < 0.001; Fig. 3). On the other hand, the absorptive surface of the medial portion of intestine was reduced only in animals receiving four doses of PVF (*F*_2,216_ = 33.95; *P* < 0.0001) (Fig. 3). Regardless of the doses administered, the distal portion showed no differences between control and treated animals (data not shown).

### Effect of PVF on carbohydrate digestion

**Figure 4:**
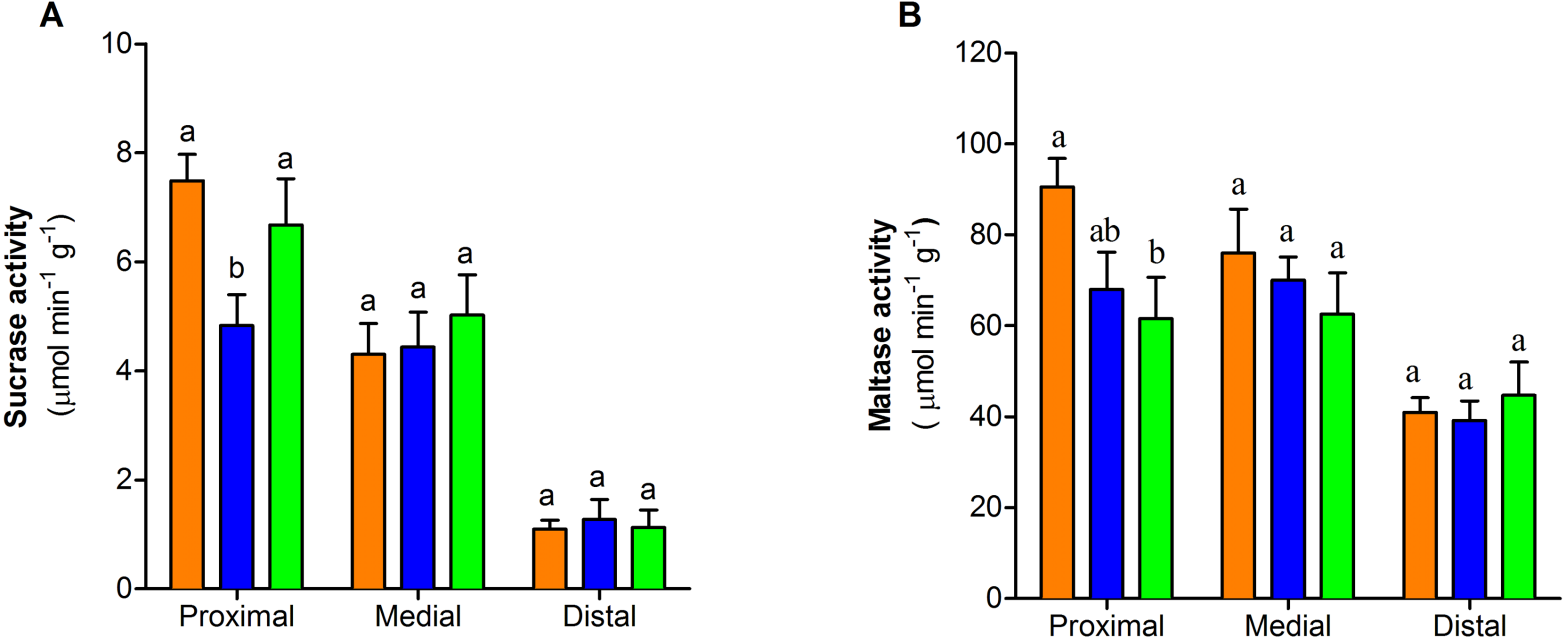
Dissaccharidase activity of the three portions of the small intestine of mice with or without PVF gavaged (A) Sucrase. (B) Maltase. Data are the mean ± SEM. Control group (orange bars), one-dose egg PVF (blue bars) and four-dose eg PVF (green bars) groups. *n* = 9 for each group. Each dose contains 0.56 mg protein.

The activity of sucrase-isomaltase decreased at the proximal section of the intestine only in one-dose group (*F*_2,25_ = 4.55, *P* < 0.05, Fig. 4A). In contrast maltase-glucoamylase activity exhibited a dose-dependent decrease at the proximal section of the intestine. (*F*_2,26_ = 3.76, *P* < 0.05, Fig. 4B). The activity of the two disaccharidases in medial and distal sections of small intestine showed no differences between control and treated groups (*P* > 0.05) (Figs. 4A–4B).

### Effect of PVF on intestine permeability

**Figure 5:**
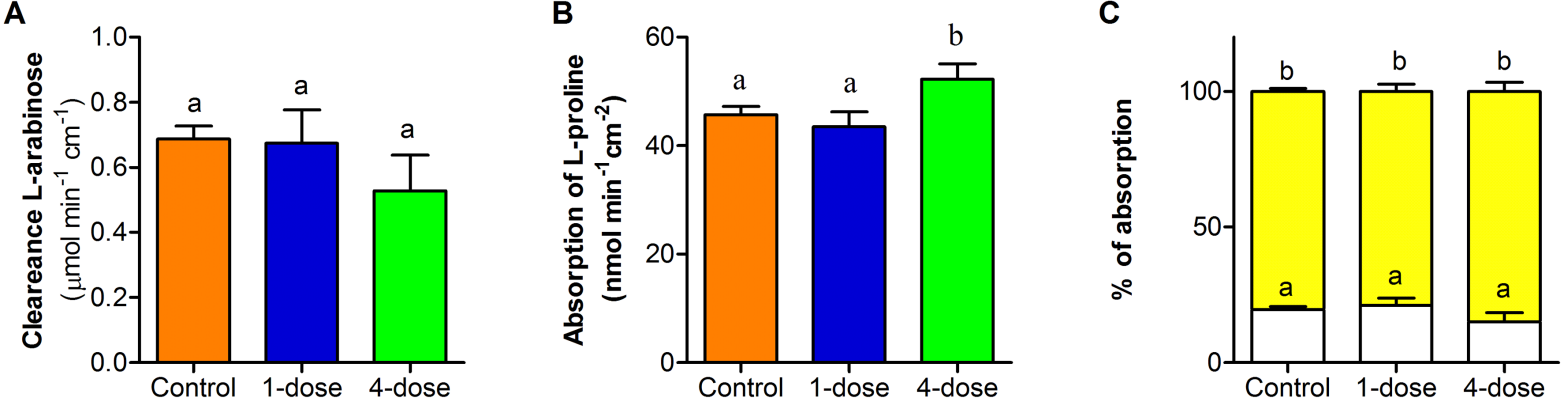
Effect of PVF from the eggs of *Pomacea maculata* on mice intestinal permeability. (A) Changes in permeability determined by the clearance of L-arabinose during a 2-h intestinal perfusion. (B) Absorption of L-proline calculated per nominal intestinal area during 2-h intestinal perfusion. (C) Relative absorption by the paracellular (white bars) and transcellular (yellow bars) pathways of L-proline, based on the absorption of L-arabinose. Control group *n* = 9; one-dose *n* = 9; four-dose *n* = 8. Data are the mean ± s.e.m; bars that share letters indicate no statistically significant difference (*P* > 0.05).

Clearance of L-arabinose shows no differences between the three groups (*F*_2,23_ = 0.96; *P* = 0.3973, Fig. 5A). The absorption of L- proline, calculated per nominal intestinal area, showed differences between control group and one-dose group in comparison to four-dose group (*F*_2,25_ = 19.95 , *P* < 0.0001, Fig. 5B). The percentage of L- proline absorption by paracellular and transcellular did not differ among groups (*F*_2,46_ = 3.07; *P* = 0.0563, Fig. 5C).

## Discussion

### Effect of the PVF on mice intestine morphology and physiology

When mice food was supplemented with *P. canaliculata* egg PVF, the gastrointestinal tract undergoes morphological changes, strongly affecting the proximal portion of the small intestine and to a lesser extent the medial portion. These changes resulted in a substantial reduction of the absorptive area of proximal and medial regions of treated animals. Similar morphological effects were seen in the proximal portion of the small intestine of rats ingesting apple snail PVF, which were also accompanied by an increased amount of goblet cells and enterocyte proliferation (Dreon et al., 2014). Moreover, similar effects on intestinal mucosa were seen in rats orally administered plant toxins such as the phytohemagglutinin toxic lectin, resulting in disturbed gut morphology with villi shortening and rapid decreases in disaccharidase activities and macromolecular absorption capacity (Bardocz et al., 1995; Linderoth et al., 2006). In fasting animals and in animals fed with a low-quality diet, comparable morphological changes are associated with energy-saving physiological adjustments to reduce the high metabolic cost of the gastrointestinal tissues (Chediack et al., 2012; Cramp, Franklin & Meyer, 2005; Thaysen & Thaysen, 1949). In this regard, several antidigestive (digestion inhibition) and antinutritive (non-digestible) compounds were identified from *Pomacea* PVF (Dreon, Ituarte & Heras, 2010; Giglio et al., 2016; Pasquevich et al., 2017) which combined provide a low-quality diet to a predator.

PVF ingestion not only adversely altered gut morphology but also impacted on intestinal physiology, particularly on the activity of some digestive enzymes (antidigestive property). Previous reports showed the PVF inhibit proteases, notably *P. canaliculata* and *P. maculata* PVFs inhibit soluble proteases secreted to the intestinal lumen such as trypsin, chymotrypsin and elastase (Dreon, Ituarte & Heras, 2010; Giglio et al., 2016). Here we found that PVF has the capacity to also inhibit membrane-bound enzymes, particularly the brush-border disaccharidases, which further extend the enzyme inhibition capacity of apple snail eggs to enzymes of the carbohydrate metabolism. A decrease in intestinal maltase and sucrase activity was also observed in rats administered with phytohemagglutinin (Linderoth et al., 2006). In our experiments, after one dose of PVF, both maltase-glucoamylase and sucrase-isomaltase diminished its activity in the proximal region of intestine in comparison with control animals.

However, while maltase-glucoamylase further diminished its activity after a longer exposure to PVF, the four-dose group restored sucrase-isomaltase activity to control levels. This differential response to stressors was also observed in rodents exposed to temperature, diet and alkaloids (Del Valle, Busch & López Mañanes, 2006; Del Valle, Mañanes & Busch, 2004; Pan, Ghidoni & Elbein, 1993). Along with morphological changes, a stronger response was observed in the proximal region while the medial and distal portions displayed a lesser or no response, respectively. This gut response has been described for other oral toxins and is most probably due to the fact that this region is the first one in contact with the ingested toxin (Gavhane & Yadav, 2012).

Animals exposed to plant dietary toxic lectins as well as fasting animals usually display a reduction of gut mucosal area which in turn, causes a reduction of nutrient absorption (Ishiguro et al., 1984; Secor, 2005b; Secor, 2005a). However, PVF effect on mice small intestine is somewhat different: although the mucosal area is also reduced, aminoacid absorption increased after a long exposure to PVF by still unknown mechanisms. The changes in mice gut morphology might be an attempt to adapt to the PVF exposure, which is possible due to gut large plasticity (Timmons et al., 2012). In addition, the increase in mucous secretion, as suggested by the increase number of goblet cells in treated mice, may be another adaptative mechanism that isolates and protects the intestinal surface from the toxic proteins. Further analysis is required to better understand this issue.

### Eco-evolutionary implications

Eggs are one of the most vulnerable life cycle stages and subjected to intense predation since they are a rich source of easy-to-obtain nutrients for predators (Dussourd et al., 1988). *Pomacea canaliculata* snails have evolved an embryo defense system that is very effective against predators since only one egg predator has been reported so far (Dreon et al., 2013; Hayes et al., 2015; Yusa, Sugiura & Ichinose, 2000). As noted previously, this results from the presence of several overlapping defenses, many targeting the digestive system of putative predators, including antinutritive compounds, enzyme inhibitors, toxic dietary lectins, and toxins affecting intestinal cells (Dreon et al., 2014; Dreon et al., 2013; Dreon, Ituarte & Heras, 2010; Giglio et al., 2016; Ituarte et al., 2012). Similar combined defenses have been found in plant seeds but as far as we know, in animals these defenses has only been reported for *Pomacea* eggs (Dreon et al., 2013; Dreon, Ituarte & Heras, 2010) and, to some extent, for the incubation foam of the túngara frog (Fleming et al., 2009). Unlike plant seeds, *Pomacea* eggs not only have noxious compounds, but their noxiousness is advertised by pigmented proteins providing a conspicuous coloration, a true warning signal preventing predators from consuming them (Heras et al., 2007; Pasquevich, Dreon & Heras, 2014).

This defensive trait may have played a role in the wide distribution, substantial evolutionary sucess, and invasiveness observed for *P. maculata* and *P. canaliculata*.

## Conclusions and Future Directions

The ingestion of PVF limits the ability of mice to digest and absorb nutrients and alters gut morphophysiology. *Pomacea canaliculata* PVF decreases gut absorptive surface, inhibits disaccharidase activity, and affects the absorption rate of nutrients in the small intestine of mice, without affecting the relative contribution of paracellular and transcellular pathways or gut permeability. These results further support the presence of passive anti-predator defenses in the eggs of *Pomacea* snails targeting more aspects of the digestive metabolism than previously thought. This defensive strategy is unparalleled in animals. It is probable that PVF lectins such as PmPV2 would be implicated in some of the observed effects. Ongoing research is looking at this question by the identification and characterization of the molecules causing these gut changes.

##  Supplemental Information

10.7717/peerj.5314/supp-1 Table S1 Individual values for mucosal to serosal ratio (*M*) Individual values for the morphology and mucosal to serosal ratio (M) for the different intestinal sections calculated using Kisielinski et al. (2002) for control mice and treated with either 1 or 4 doses (each dose = 560 µg protein) of perivitelline fluid of the eggs of *Pomacea canaliculata.*

10.7717/peerj.5314/supp-2 Table S2 Intestinal disaccharidases activities

10.7717/peerj.5314/supp-3 Table S3 Absorption values

## Additional Information and Declarations

### Data Availability

The following information was supplied regarding data availability: The raw data are provided in the Supplemental Files.
